# Polydopamine-Functionalized Copper Peroxide/ZIF-8 Nanoparticle-Based Fluorescence-Linked Immunosorbent Assay for the Sensitive Determination of Carcinoembryonic Antigen by Self-Supplied H_2_O_2_ Generation

**DOI:** 10.3390/bios12100830

**Published:** 2022-10-06

**Authors:** Juanjuan Huang, Yiyun Yao, Yanling Chen, Tianran Lin, Li Hou, Dianping Tang

**Affiliations:** 1 School of Chemistry and Pharmaceutical Sciences, State Key Laboratory for Chemistry and Molecular Engineering of Medicinal Resources, Guangxi Normal University, Guilin 541004, China; 2 Key Laboratory of Analytical Science for Food Safety and Biology (MOE & Fujian Province), Department of Chemistry, Fuzhou University, Fuzhou 350108, China

**Keywords:** fluorescence-linked immunosorbent assay, Fenton-type reaction, carcinoembryonic antigen

## Abstract

Copper peroxide/zeolitic imidazolate framework/polydopamine nanoparticles (CP/ZIF-8/PDA)-based fluorescence-linked immunosorbent assay (FLISA) was designed for the sensitive and high-throughput determination of carcinoembryonic antigen (CEA) by self-supplied H_2_O_2_ generation. Specifically, the CEA aptamer was modified on the surface of CP/ZIF-8/PDA to form an immunoprobe. The structures of CP and ZIF-8 could be broken under acidic conditions, and produced the Cu^2+^ and H_2_O_2_ due to the dissociation the CP. A subsequent Fenton-type reaction of Cu^2+^ and H_2_O_2_ generated hydroxyl radical (·OH). o-phenylenediamine (OPD) was oxidized by the ·OH to form 2, 3-diaminophenazine (DPA) with a significant fluorescence signal. CP/ZIF-8/PDA could be used as an efficient Fenton-type reactant to generate a large amount of ·OH to promote OPD oxidation. The sensitive detection of CEA could be realized. Under optimal conditions, the FLISA platform displayed a linear detection range from 0.01 to 20 ng mL^−1^ with a detection limit of 7.6 pg mL^−1^ for CEA. This strategy has great application potential for sensitive and high-throughput determination for other biomarkers in the field of biomedicine.

## 1. Introduction

The enzyme-linked immunosorbent assay (ELISA) is the gold standard for the detection of a large number of routine biological analytes [1]. Nevertheless, conventional ELISA still suffers from high background, low sensitivity, false-positive results, it is expensive and the labeling enzyme is highly susceptible to inactivation [2,3], so the practical applications of the ELISA technique is restricted. Normally, enzyme-labeled secondary antibodies are need for ELISA to determine the absorbance of the substrate [4]. Distinct from the ELISA, fluorescence-linked immunosorbent assay (FLISA) employs a fluorescent substance as a label to measure the fluorescence intensity [5]. Both ELISA and FLISA are efficient high-throughput detection methods. The principles of their operation and detection are similar. The rapid development of nanotechnology has made tremendous progress in the development of the FLISA [6]. In recent years, many fluorescent materials have been developed for the FLISA method, including graphene oxide (GO) [7] and metal-organic frameworks [8]. These materials generally use the fluorescence resonance energy transfer (FRET) strategy to achieve a sensitive signal output, but the donor and acceptor of FRET are susceptible to distance and depend on the degree of overlap between the emission spectrum of the donor and the excitation spectrum of the acceptor. In addition, many signal amplification methods of enhancing the sensitivity of FLISA have been investigated, including carbon dots (o-CDs) aggregation fluorescence quenching [9], the simple enhancement of fluorescence by Triton-X 100 micelles [10] and signal amplification by fluorescein-labeled DNA strand hybridization reaction [11]. However, these methods have limitations for analytical applications, such as their susceptibility to interference by metal ions and the high cost of reagent. Therefore, the exploration of the fluorescence output strategy and appropriate nanomaterial in FLISA are still very meaningful.

Zeolitic imidazolate frameworks (ZIFs) are typical materials for metal organic frameworks (MOFs), which have been widely used in sensors and drug delivery due to their large specific surface area, good porosity and biocompatibility [12,13]. Because of the high affinity of ZIFs for enzymes, Guo et al. [14] designed a sensor based on the enzymatic cascade reaction of metal-organic framework composites (AuNCs/β-Gal/GOx@ZIF-8) for the detection of lactose. The sensitivity of this sensor was effectively enhanced by catalytic signal amplification. However, the biological enzymes are expensive and easily lose activity since they are susceptible to external conditions, including temperature and pH changes, as well as a complicated modification process. Therefore, enzyme-mediated catalytic signal amplification by assembling enzyme-ZIFs nanocomposites is not an ideal signal output strategy. Wang et al. [15] synthesized a copper peroxide/zeolitic imidazolate framework/polydopamine (CP/ZIF-8/PDA) composite, which was combined with a 3-aminobenzeneboronic acid (ABA)/polyvinyl alcohol (PVA) film-modified electrode and self-supplied H_2_O_2_ generation for the electrochemical immunoassay of the glycoantigen CA19-9. This method used HCl-triggered cascade reaction without introducing enzymes and metal ions, which had good sensitivity, selectivity and stability, and provided a new idea for designing simple, fast and effective immunosensors. Above all, the known ZIFs-based FLISA for the sensitive and high-throughput determination of CEA by self-supplied H_2_O_2_ generation has not reported so far.

**Scheme 1:**
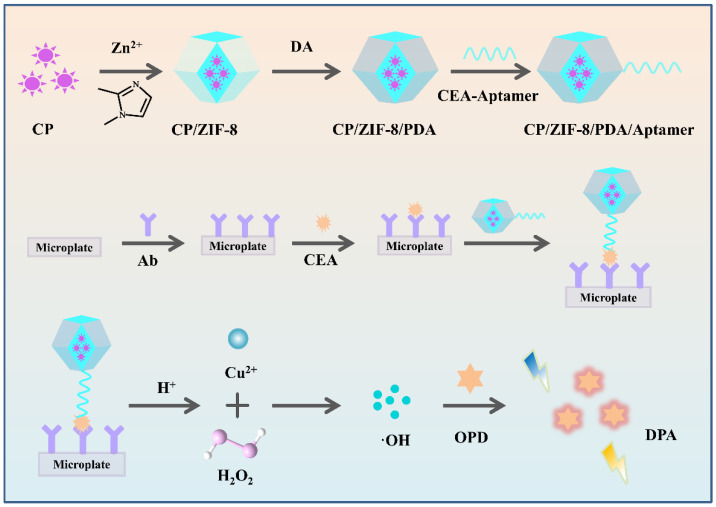
Schematic illustration of CP/ZIF-8/PDA nanoparticle-based FLISA for the sensitive determination of the carcinoembryonic antigen by self-supplied H_2_O_2_ generation.

Inspired by the above work, a FLISA platform for the sensitive and high-throughput detection of the carcinoembryonic antigen (CEA) was constructed based on a CP/ZIF-8/PDA nanocomposite by self-supplied H_2_O_2_ generation (Scheme 1). ZIF-8 was used for the encapsulation of CP for the formation of ZIF-8/CP. After a layer of PDA membrane was formed onto the surface of ZIF-8/CP, the abundant amino of PDA was beneficial to the immobilization of biomolecules, such as antibodies or aptamers via the EDC-NHS reaction and glutaraldehyde cross-linking reaction. CP was the source of Cu^2+^ and H_2_O_2_ under the stimulation of the acidic condition. Thus, the CP/ZIF-8/PDA plays a key role as a “signal switch” and immunocarrier. The aminated CEA aptamer was modified on the surface of CP/ZIF-8/PDA to form an immunoprobe (CP/ZIF-8/PDA/Aptamer) via the glutaraldehyde cross-linking function between the aminated CEA aptamer and amino-rich PDA. In the presence of CEA, CP/ZIF-8/PDA/Aptamer and the modified CEA antibodies on the microtiter plate specifically recognized with CEA to form the sandwich immune complexes. Then, the structure of CP/ZIF-8/PDA was destroyed under acidic conditions, thus, releasing CP, which was rapidly converted to Cu^2+^ and H_2_O_2_ [16]. After that, Cu^2+^ underwent a Fenton-type reaction with H_2_O_2_ to produce ·OH and ·OH-oxidized o-phenylenediamine (OPD) to form 2,3-diaminophenothiazine (DPA) with fluorescence property. This method did not involve enzymes, metal ions and external H_2_O_2_ to induce Fenton-type reactions. Compared with an external H_2_O_2_-based sensing system, self-supplied H_2_O_2_ was more stable in the system solution and could effectively avoid the reduction in biomolecular activity [17]. The simultaneous production of H_2_O_2_ and Cu^2+^ could achieve enrichment in site to form higher concentrations of H_2_O_2_ and Cu^2+^, which was conducive to producing a large amount of ·OH to oxidize OPD. The efficient high-throughput detection of CEA could be achieved by the FLISA sensing platform. Therefore, polydopamine-functionalized CP/ZIF-8 nanoparticle-based FLISA provided a new strategy for the sensitive and high-throughput detection of CEA.

## 2. Experimental Section

### 2.1. Reagents and Materials

We purchased dopamine (DA), copper chloride dihydrate (CuCl_2_·2H_2_O), glacial acetic acid (HAc), sodium acetate (NaAc), sodium carbonate (Na_2_CO_3_), sodium bicarbonate (NaHCO_3_), sodium dihydrogen phosphate (Na_2_HPO_4_) and disodium hydrogen phosphate (Na_2_HPO_4_) from Aladdin Reagent Co., Ltd. (Shanghai, China). Solarbio Science & Technology. Co., Ltd. (Beijing, China) provided bovine serum albumin (BSA, 97.0%) and Tween 20. From Jiuding Chemical Technology Co., Ltd. (Shanghai, China), we purchased methylimidazole and o-Diphenylamine (OPD). Sodium chloride (NaCl), potassium chloride (KCl) and sodium hydroxide (NaOH) were purchased from Sinopharm Chem. Re. Co., Ltd. (Shanghai, China). Zn(NO_3_)_2_·6H_2_O and anhydrous methanol were acquired from Xilong Scientific Co., Ltd. (Guangdong, China). All other chemicals were analytical grade. In these experiments, ultrapure water (Milli-Q, Millipore, ≥18.2 MΩ·cm) was used. Sangon Biotech (Shanghai, China) supplied the high-performance liquid chromatography (HPLC) purified CEA-binding aptamer. The following oligonucleotide sequence (5’ → 3’) was listed: H_2_N-TTT TAT ACC AGC TTA TTC AAT T. Beijing Key-Bio Biotech Co., Ltd. (Beijing, China) provided the CEA monoclonal antibody (5 mg mL^−1^), CEA (100 μg mL^−1^), CA19-9 (50 KIU mL^−1^), CA125 (50 KIU mL^−1^), AFP (105 μg mL^−1^) and PSA (100 μg mL^−1^). The Guilin Hospital of Chinese Traditional and Western Medicine provided clinical human serum samples with different CEA concentrations based on the rules of the local ethical committee. The referenced values of CEA in clinical human serum samples were acquired from the commercialized Roche Cobas e601 automatic electrochemiluminescence immunoanalyzer (Basel, Switzerland)

### 2.2. Apparatus

Perkin-Elmer (USA) provided Fourier transform infrared (FTIR) spectrometer. Thermo Fisher (Thermo Fisher Scientific, Waltham, MA, USA) supplied field emission transmission electron microscopy (FE-TEM). The sample shaking was performed by Mini Shaker (Kylin-Bell Lab Instruments Co., Ltd., Haimen, China). Mapada (Shanghai, China) supplied the ultraviolet-vis absorption UV-6100S spectrophotometer. Malvern (Malvern, UK) supplied the Zetasizer Nano ZS90 Laser Particle Size/Potentiometer. Tecan (Männedorf, Switzerland) provided the TECAN microplate reader SPARK. The emission spectra at 565 nm wavelength were applied with the excitation spectra of DPA at 410 nm.

### 2.3. Synthesis of CP/ZIF-8/PDA

CP nanoparticles were prepared according to the literature synthesis method [16]. A total of 0.5 g of polyvinylpyrrolidone (PVP) and 0.01 g of CuCl_2_·2H_2_O were dissolved in 5 mL of aqueous solution. Then, 0.1 mL of H_2_O_2_ (30%) and 5 mL of NaOH (0.02 mol L^−1^) were added to the above solution, and the color of the solution was changed from clear to brown by magnetic stirring for 30 min. The PVP-coated CP nanoparticles were washed by multiple centrifugations (12,000 rpm) with ultrapure water, and the centrifuged CP was dispersed with anhydrous methanol and stored in a refrigerator at 4 °C for use.

A total of 291 mg of Zn(NO_3_)_2_·6H_2_O and 328 mg of 2-methylimidazole were sequentially dispersed in 25 mL of CP solution, stirred magnetically at room temperature for 24 h. CP/ZIF-8 was obtained by centrifugal washing (8000 rpm) and dried at 60 °C for 12 h.

A total of 100 mg of CP/ZIF-8 was weighed and dispersed in 25 mL of methanol. Subsequently, 10 mL of dopamine solution (DA, 1.0 mg mL^−1^, dissolved in Tris-HCl, pH 8.5) was added under magnetic stirring and the reaction was continued for 2.5 h to form a polydopamine film (PDA) on the surface of CP/ZIF-8. CP/ZIF-8/PDA was obtained by centrifugal washing (8000 rpm) and dried at 60 °C for 12 h.

### 2.4. Synthesis of the CP/ZIF-8/PDA/Aptamer Immunoprobe

A total of 20 mg of CP/ZIF-8/PDA was dispersed into 20 mL of PBS buffer (10 mmol L^−^^1^, pH 7.4), and 1 mL of 50% glutaraldehyde was added. The above resulting solution was incubated in a shaker at room temperature for 2 h, and the surface-activated CP/ZIF-8/PDA was washed by centrifugation at 10,000 rpm for 3 times and dispersed in 6.5 mL of PBS. Then, 0.5 OD of aminated CEA Aptamer was added and shaken at 37 °C for 16 h. After that, the immunoprobe (CP/ZIF-8/PDA/Aptamer) was obtained by adding BSA (final concentration of 1%) and shaking at room temperature for 1 h. The immunoprobe (CP/ZIF-8/PDA/Aptamer) was finally washed by centrifugation at 9500 rpm.

### 2.5. The 96-Well Plate Trimmed with CEA Antibody

First, the CEA antibody was diluted to 10 µg mL^−1^ with Na_2_CO_3_-NaHCO_3_ coating buffer (100 mmol L^−1^, pH 9.6). A total of 100 µL of CEA antibody was then added to each well of a 96-well plate and incubated for 12 h at 4 °C in the refrigerator. Then, the well plates were washed for three times with PBST wash buffer (10 mmol L^−^^1^, pH 7.4, 27 mmol L^−1^ KCl, 135 mmol L^−1^ NaCl, 0.05% Tween 20), and then 200 µL of PBST buffer containing 1% BSA as blocking agent was added and incubated for 1 h at room temperature to block the remaining active sites. The above washing operation was repeated, and finally the 96-well plate was sealed with cling film and stored in a refrigerator at 4 °C.

### 2.6. FLISA Determination of CEA

A total of 200 µL of different concentrations of CEA were added to the microtiter wells and incubated at 37 °C for 1 h. Excess CEA was washed off with PBS (10 mmol L^−1^, pH 7.4). Next, 100 µL of CP/ZIF-8/PDA/Aptamer immunoprobe was added and incubated at room temperature for 1 h. Then, excess immunoprobe was washed with PBS. Then, 100 µL of 4 mmol L^−1^ OPD (prepared with pH 5.5, 10 mmol L^−1^ HAc-NaAc buffer solution) was added for the reaction of 3 h. The fluorescence signal intensity at 565 nm was measured by a TECAN microplate reader SPARK.

## 3. Results and Discussion

### 3.1. Characterization of the CP and CP/ZIF-8/PDA/Aptamer

**Figure 1:**
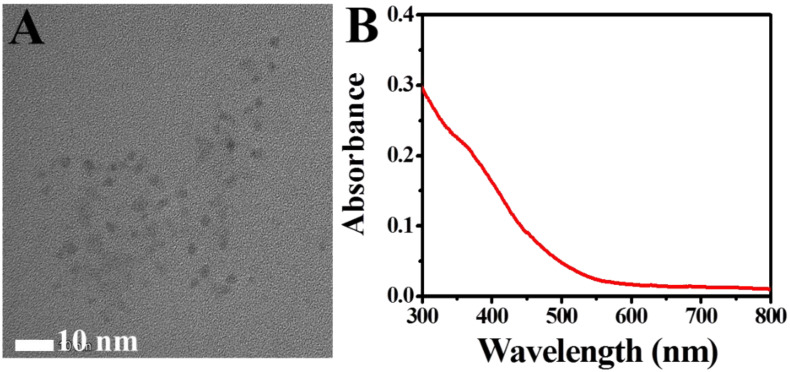
(**A**) TEM images of CP and (**B**) UV absorption peaks of CP.

The morphology of the synthesized CP nanoparticles was characterized by TEM (Figure 1A). As seen in Figure 1A, the CP nanoparticles are small, spherical particles with a size of about 3 nm and uniform distribution. Figure 1B shows the UV-vis of CP nanoparticles in the aqueous solution; it can be seen that there is a shoulder peak at 345 nm, which is an absorption peak due to the charge transfer between O^2-^ and Cu(II) through a symmetrical bridging structure [18].

**Figure 2:**
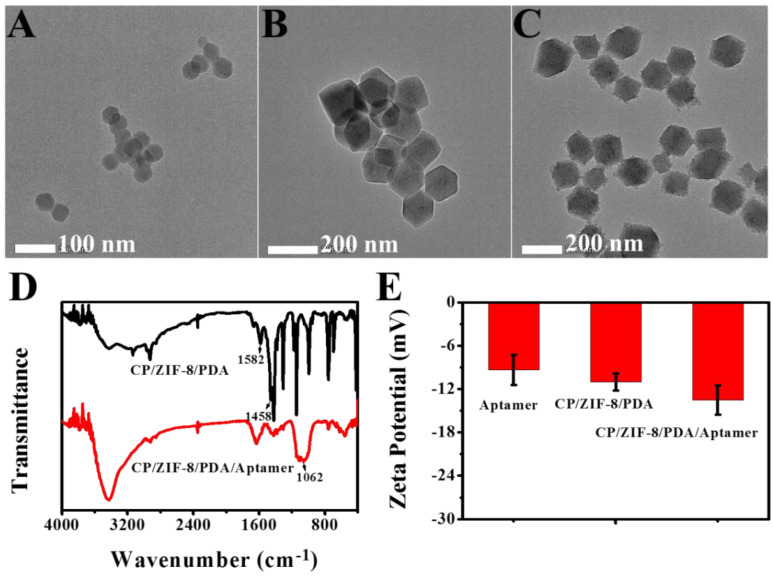
TEM image of (**A**) ZIF-8, (**B**) ZIF-8/CP, (C) CP/ZIF-8/PDA, and (**D**) FT-IR spectra of CP/ZIF-8/PDA, CP/ZIF-8/PDA/Aptamer; (**E**) Zeta potentials of CEA Aptamer, CP/ZIF-8/PDA and CP/ZIF-8/PDA/Aptamer.

Energy dispersive spectrometric spectra for the TEM images (EDS) were performed for ZIF-8 and CP/ZIF-8, respectively. Among them, Figure S1 shows the EDS mapping element analysis diagram of ZIF-8, from which ZIF-8 contained four elements, C, N, Zn and O. In contrast to Figure S2, the EDS mapping elemental analysis plot of CP/ZIF-8 showed that CP/ZIF-8 contained Cu elements in addition to the above four elements, indicating that CP nanoparticles were successfully encapsulated into ZIF-8. TEM of ZIF-8, CP/ZIF-8 and CP/ZIF-8/PDA were also performed (Figure 2) to examine the morphology of the materials after modification. In Figure 2A and B, after the CP nanoparticles were covered with ZIF-8, many CP nanoparticles were distributed in ZIF-8, and the shadow in the middle of the TEM image deepened. Additionally, the particle size of the nanoparticles became larger with about 150–200 nm, further indicating the successful synthesis of CP/ZIF-8 nanoparticles. With the coverage of PDA, the surface of CP/ZIF-8 nanoparticles gradually became rough (Figure 2C), and the shadow deepened again, indicating the successful modification of PDA on the surface of CP/ZIF-8.

The successful synthesis of CP/ZIF-8/PDA/Aptamer can be verified by FTIR. In Figure 2D, it could be seen that CP/ZIF-8/PDA had significant absorption bands at 1458 cm^−1^and 1582 cm^−1^, which were caused by the stretching vibrations of the aromatic ring skeleton in PDA [19], indicating the presence of PDA on the surface of CP/ZIF-8. Two absorption bands were observed between 3200–3500 cm^−1^, which were attributed to the N-H stretching vibrations of -NH_2_ on the PDA. When the CP/ZIF-8/PDA was modified with the aptamer by glutaraldehyde cross-linking method, the double peak between 3200–3500 cm^−1^ became a single peak, which was due to the N-H stretching vibration peak formed by glutaraldehyde crosslinking to produce amide bond, and -NH_2_ became -NH. A clear absorption band at 1062 cm^−1^ appeared in the FTIR spectrum of CP/ZIF-8/PDA/Aptamer, which was mainly caused by the phosphodiester bond in the DNA strand [20], indicating the successful synthesis of CP/ZIF-8/PDA/Aptamer. Meanwhile, the zeta potentials of aptamer, CP/ZIF-8/PDA and CP/ZIF-8/PDA/Aptamer were also examined, as shown in Figure 2E. The average zeta potential carried by the CEA Aptamer was −9.36 mV and that of CP/ZIF-8/PDA was −11.02 mV. When CP/ZIF-8/PDA was modified with CEA Aptamer, the average zeta potential was −13.57 mV. These results further demonstrate the successful synthesis of CP/ZIF-8/PDA/Aptamer.

### 3.2. Feasibility of the Proposed FLISA Platform

**Figure 3:**
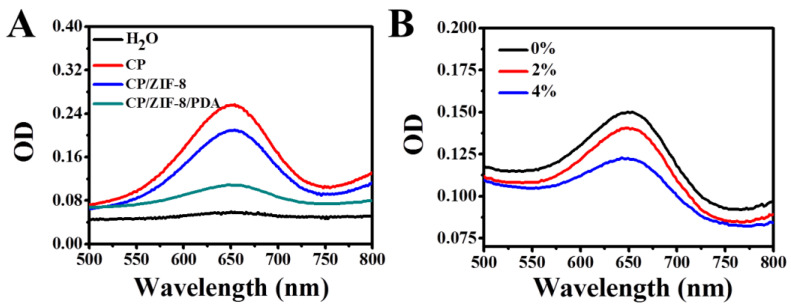
(**A**) Investigation of the catalytic oxidation of TMB by H_2_O, CP, CP/ZIF-8, CP/ZIF-8/PDA, (**B**) investigation of ·OH by tert-butanol as a scavenger (0/2/4% is the volume ratio of tert-butanol in the solution).

CP nanoparticles can be decomposed to form Cu^2+^ and H_2_O_2_ under acidic conditions, and Cu^2+^ and H_2_O_2_ will produce ·OH through the Fenton-type reaction. To verify the ability of CP/ZIF-8/PDA to catalyze the oxidation of 3,3’,5,5’-tetramethylbenzidine (TMB), H_2_O, CP, CP/ZIF-8/and CP/ZIF-8/PDA were reacted with TMB under acidic conditions for 10 min. As can be seen in Figure 3A, under acidic conditions, H_2_O cannot oxidize TMB with almost no UV absorption peak, while all CP, CP/ZIF-8 and CP/ZIF-8/PDA can oxidize TMB with different degrees of UV absorption peaks at 652 nm. The results indicate that CP still had the ability to catalyze the oxidation of TMB through the encapsulation and modification of ZIF-8 and PDA to produce ·OH. To further verify the generation of ·OH by CP/ZIF-8/PDA under acidic conditions, tert-butanol was used as a scavenger to react with CP/ZIF-8/PDA and TMB to identify ·OH. As shown in Figure 3B, the intensity of the UV-visible absorption peak decreases continuously with the increase in the tert-butanol concentration. The results indicate that CP/ZIF-8/PDA can generate ·OH under acidic conditions. Additionally, the fluorescence intensity of the FLISA platform after the introduction of CP/ZIF-8/PDA/Aptamer in the presence and absence of CEA was probed using OPD as the substrate (Figure S3). Comparing the absence of CEA, the fluorescence signal of the FLISA-sensing platform became significantly stronger in the presence of CEA. The above results show that the experiment is feasible.

### 3.3. Effect of Reaction Parameters

**Figure 4:**
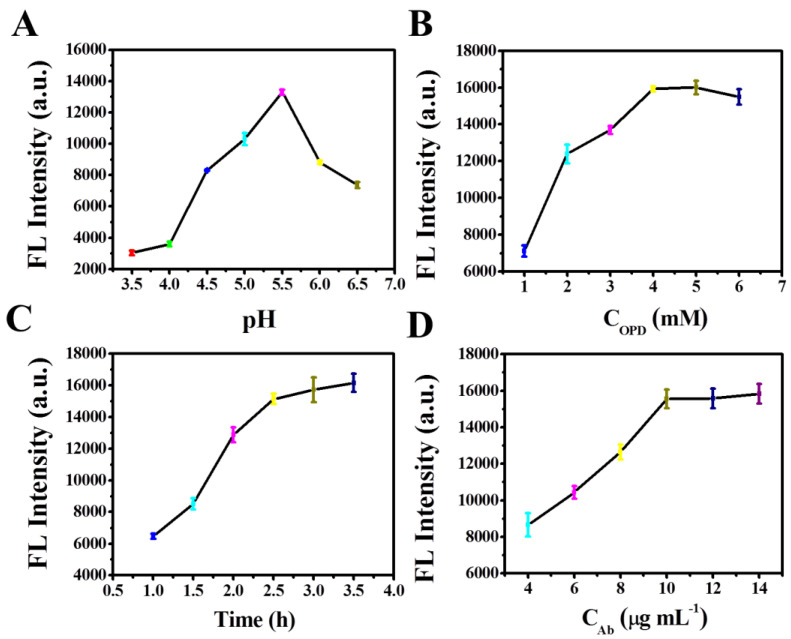
Effects of (**A**) pH of CP/ZIF-8/PDA reaction with OPD, (**B**) OPD concentration, (**C**) incubation time of OPD and (**D**) Ab concentration on the fluorescence intensity. A total of 2 mmol L^−1^ of OPD was used for pH optimization, 4 mmol L^−1^ of OPD was used for other optimization, and 5 ng mL^−1^ of CEA was used.

To obtain the optimal analytical performance of the FLISA platform, the influence of the pH value of OPD reaction, OPD concentration, incubation time of OPD and antibody concentration on the fluorescence signal were investigated, respectively. A concentration of 5 ng mL^−1^ CEA was used as the target concentration. The CP in CP/ZIF-8/PDA requires acidic release to produce ·OH to oxidize OPD, so the pH of the reaction is particularly important for the reaction system. HAc-NaAc buffer was used to dissolve OPD, and the pH of the buffer was optimized. The results in Figure 4A show that, in the pH range of 3.5 to 5.5, as the pH increased, the fluorescence signal was continuously enhanced, mainly because the oxidation of OPD was inhibited by over-acidic conditions [21]. In the pH 5.5–6.5 interval, the fluorescence signal continuously decreased with increasing pH, indicating that the dissolution of the ZIF-8 and PDA shell layers encapsulating CP slowed down after the pH reached 5.5, so pH 5.5 was chosen as the optimal pH. OPD concentration and reaction duration also have a large effect on the fluorescence signal. As shown in Figure 4B, as the OPD concentration increased from 1 mmol L^−^^1^ to 4 mmol L^−1^, the DPA formed by ·OH oxidation of OPD increased and the fluorescence signal intensity of the reaction was enhanced. The fluorescence signal leveled off after the OPD concentration reached 4 mmol L^−1^, indicating that the reaction reached equilibrium, thus, 4 mmol L^−1^ was chosen as the optimal OPD.

In Figure 4C, as the reaction time increased from 1 to 2.5 h, DPA increased, and the fluorescence intensity kept getting stronger. The fluorescence signal slowly enhanced with time growth after 2.5 h, thus, 2.5 h was chosen as the best reaction duration. Finally, the concentration of modified antibody (Ab) on the 96-well plate was optimized. As shown in Figure 4D, the concentration of Ab increased from 4 µg mL^−1^ to 10 µg mL^−1^, the CEA captured by Ab kept increasing and the more CP/ZIF-8/PDA fixed onto the well plate, thus, the intensity of the fluorescence signal obtained was greater. When the concentration of Ab reached 10 µg mL^−1^, the fluorescence signal reached a plateau, indicating that the amount of antibody binding to CEA reached saturation. Finally, 10 µg mL^−1^ of Ab was selected as the optimal concentration.

### 3.4. Analytical Performance

**Figure 5:**
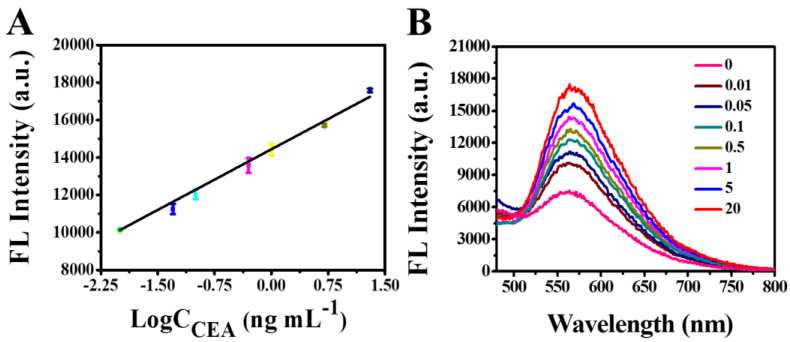
(**A**) The linear calibration plot for CEA determination (0.01–20 ng mL^−1^, log C vs. fluorescence intensity at 565 nm) (error bars: SD, *n* = 3), (**B**) fluorescent determination of CEA based on CP/ZIF-8/PDA nanoparticles by self-supplied H_2_O_2_ (the concentration of CEA: 0, 0.01, 0.05, 0.1, 0.5, 1, 5, 10 and 20 ng mL^−1^).

To examine the applicability of the FLISA analytical method for the sensitive detection of CEA, different concentrations of CEA were detected under optimal experimental conditions. In Figure 5A, the fluorescent signal was directly proportional to the logarithmic value of CEA concentrations in the range from 0.01 ng mL^−1^ to 20 ng mL^−1^, corresponding with Figure 5B. The linear regression equation was *F* = 2154.8 × l g *C*_[CEA]_/ng mL^−1^ + 14439.75 (R^2^ = 0.9967). The detection limit was as low as 7.6 pg mL^−1^ (3*σ/K*, where *σ* stands for the standard deviation of 10 blank controls, *K* stands for the slope of regression equation). The analytical performances of other CEA assays [22,23,24,25,26,27,28] were listed in Table S1 and compared with our designed ELISA method. The results showed that the sensitivity of this FLISA method was comparable or even better than other analytical methods. This is because the sensing platform triggers a cascade reaction under acidic conditions to amplify the detection signal, and the simple steps eliminate many unnecessary errors. In addition, the self-supplied H_2_O_2_ is more stable and reacts more efficiently with Cu^2+^ than the applied H_2_O_2_. This FLISA platform has a low detection limit and can be used as an effective method for the clinical diagnosis of CEA.

### 3.5. Selectivity and Stability Investigation

**Figure 6:**
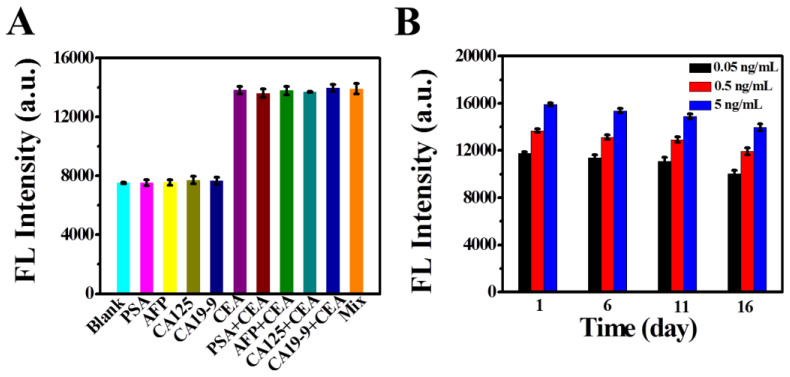
(**A**) Selectivity for the determination of CEA by monitoring the fluorescence intensity with different interfering species (error bars: SD, *n* = 3), (**B**) the stability of the FLISA platform for CEA (0.05, 0.5, 5 ng mL^−1^) (error bars: SD, *n* = 3).

To examine the selectivity of this FLISA platform for CEA, coexisting substances that may interfere with CEA determination in actual samples such as prostate antigens (PSA, 25 ng mL^−1^), alpha-fetoprotein (AFP, 25 ng mL^−1^), glycoantigen 125 (CA125, 25 IU mL^−1^) and glycoantigen 19-9 (CA19-9, 25 IU mL^−1^) were investigated. The fluorescence signal intensity of 0.5 ng mL^−1^ CEA and interfering antigens and their mixtures were detected, respectively. The results are shown in Figure 6A. When comparing with other interferents, it was found that the fluorescence signal intensity of the experimental group with CEA was larger, and the fluorescence signal intensity of the interferent alone was consistent with that of the blank group. This was because the fluorescence immunosensor could not recognize other coexisting substances. When the CEA and high concentration of other tumor markers coexist in the sample, the fluorescence response results were in agreement with the results from the single presence of CEA. These results demonstrated that the fluorescence immunosensor could specifically distinguish CEA from its interfering species since the fluorescence signal was specifically triggered by the binding of the aptamer/antibody and CEA. So, the interferents did not interfere with the detection process. The results showed that the analytical method had good specificity for CEA. The reproducibility of the analytical method was investigated at three different concentrations of CEA. The coefficients of variation (CVs) of the three intra-batch measurements were 2.13%, 1.38% and 1.21% for the three CEA concentrations of 0.05, 0.5 and 5 ng mL^−1^, respectively, and the CVs of the three inter-batch measurements were 5.09%, 6.10% and 6.17%, respectively. The results indicate that the FLISA platform has good reproducibility. The stability of the fluorescent immunosensor is a key factor which affects the practical application. As shown in Figure 6B, when the CP/ZIF-8/PDA/Aptamer was stored at 4 °C for 1, 6, 11 and 16 days, respectively, for the same concentration of CEA, the fluorescence signal change was not obvious. However, for the different concentrations of CEA, with the increasement in CEA concentration from 0.05 ng mL^−1^, 0.5 ng mL^−1^ to 5 ng mL^−1^, the fluorescence signal intensity gradually increased, which was consistent with the result of the linear calibration plot for CEA determination. The above results indicate that the method has good stability.

### 3.6. Analysis of Real Samples and Evaluation of Method Accuracy

The CEA referenced the concentrations of six clinical serum samples, which were 0.05, 0.5, 1.5, 2.1, 5.4 and 13.7 ng mL^−1^ for the follow-up FLISA determination, respectively. Among them, serum samples containing 0.05 ng mL^−1^ and 13.7 ng mL^−1^ CEA were obtained via dilution with PBS (10 mmol L^−1^, pH 7.4). In Table 1, the FLISA results of the six different samples were consistent with the referenced values obtained from the commercialized Roche Cobas e601 automatic electrochemiluminescence immunoanalyzer. The maximum RSD of the FLISA method did not exceed 8.16%, indicating that the method has high accuracy and clinical application.

## 4. Conclusions

In summary, a self-supplied H_2_O_2_ of FLISA method was constructed based on CP/ZIF-8/PDA nanoparticles for the sensitive and high-throughput determination of CEA. The prepared CP/ZIF-8/PDA nanoparticles were modified by the aptamer to form an immunoprobe and were immobilized onto a 96-well plate by forming a sandwich complex with CEA and antibodies. Under acidic conditions, the cascade reaction was triggered to generate Cu^2+^ and H_2_O_2_ due to the CP dissociation. Both of them rapidly underwent a Fenton-type reaction to produce ·OH which oxidized OPD to form a strong fluorescent substance DPA for subsequent fluorescence detection. Because of the close distance between the simultaneously generated Cu^2+^ and H_2_O_2_, the concentration of them in the site was relatively high, and thus, a high reaction efficiency could be achieved without the addition of H_2_O_2_. Additionally, the signal output of this FLISA platform did not require enzymes. The sensitive determination of CEA could be achieved with a LOD value of 7.6 pg mL^−1^, and the result of CEA determination in serum samples was satisfactory. Thus, the FLISA-sensing platform provided a new way of sensitive and large-scale screening biomarkers for the early diagnosis of clinical disease. Future work will focus on the point of care diagnosis for CEA via smartphone determination and explore app applications towards the biomarker-based FLISA method.

## Figures and Tables

**Table 1:** Comparison of the results for the determination of CEA in clinical serum samples by using the self-supplied H_2_O_2_ fluorescent immunosensor and the referenced electrochemiluminescence method.

Sample Number	Found by the Electrochemiluminescence Method [ng mL^−1^]	Found by Self-Supplied H_2_O_2_ Fluorescent Immunosensor [mean ± SD (RSD), ng mL^−1^], *n* = 3
1	0.05	0.052 ± 0.001 (2.54%)
2	0.5	0.51 ± 0.03 (5.84%)
3	1.5	1.55 ± 0.13 (8.16%)
4	2.1	1.99 ± 0.14 (7.20%)
5	5.4	5.94 ± 0.17 (5.24%)
6	13.7	13.05 ± 0.68 (5.24%)

## Data Availability

Not applicable.

## Supplementary Materials

The following supporting information can be downloaded at: https://www.mdpi.com/article/10.3390/bios12100830/s1, Figure S1: EDS mapping element analysis diagram of ZIF-8; Figure S2: EDS mapping element analysis diagram of CP/ZIF-8; Figure S3: Fluorescence intensity of FLISA platform with and without CEA; Table S1: Comparison of analytical performance for CEA determination by using different sensing methods.
