# Supplementary material for: Polydopamine-Functionalized Copper Peroxide/ZIF-8 Nanoparticle-Based Fluorescence-Linked Immunosorbent Assay for the Sensitive Determination of Carcinoembryonic Antigen by Self-Supplied H2O2 Generation
Source: Biosensors (Basel). 2022 Oct 6;12(10):830. doi: 10.3390/bios12100830 (PMC9599728; doi:10.3390/bios12100830)
Supplement: Supplementary file 1 [file biosensors-12-00830-s001.zip › biosensors-1923479-supplementary.pdf]

biosensors

Electronic Supplementary Materia

Polydopamine-Functionalized Copper Peroxide/ZIF-8 Nanoparticles-Based Fluorescence-Linked Immunosorbent Assay for Sensitive Determination of Carcinoembryonic Antigen by Self-Supplied H_2O_2 Generation

Juanjuan Huang [1,†], Yiyun Yao [1,†], Yanling Chen [1], Tianran Lin [1], Li Hou [1,*] and Dianping Tang [2,*]

Table of Contents:

1. Supporting Figures and Tables:

Figure S1. EDS mapping element analysis diagram of ZIF-8.
Figure S2. EDS mapping element analysis diagram of CP/ZIF-8.
Figure S3. Fluorescence intensity of FLISA platform with and without CEA.
Table S1. Comparison of analytical performance for CEA determination by using different sensing methods.

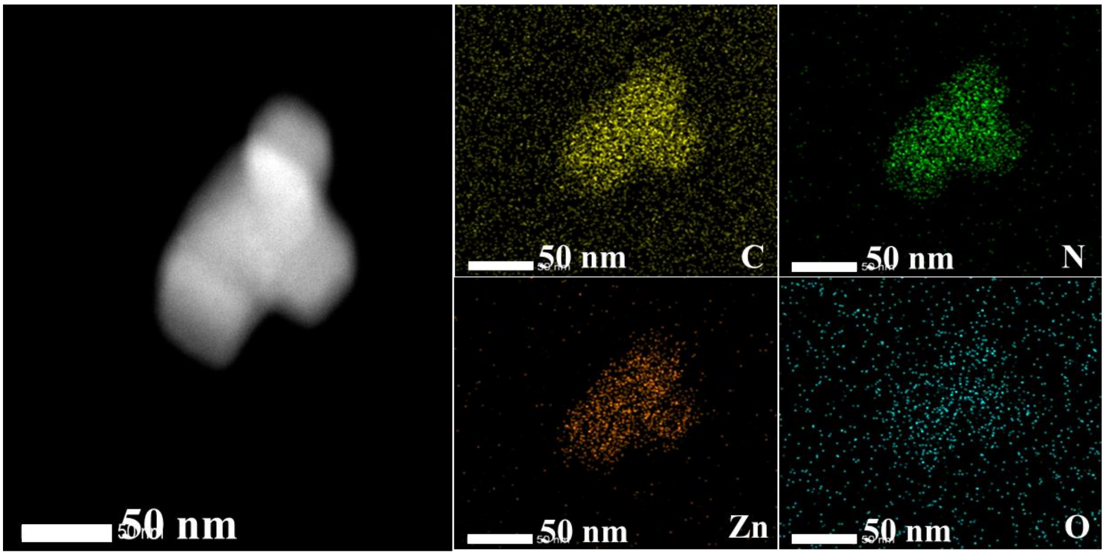

Figure S1. EDS mapping element analysis diagram of ZIF-8.

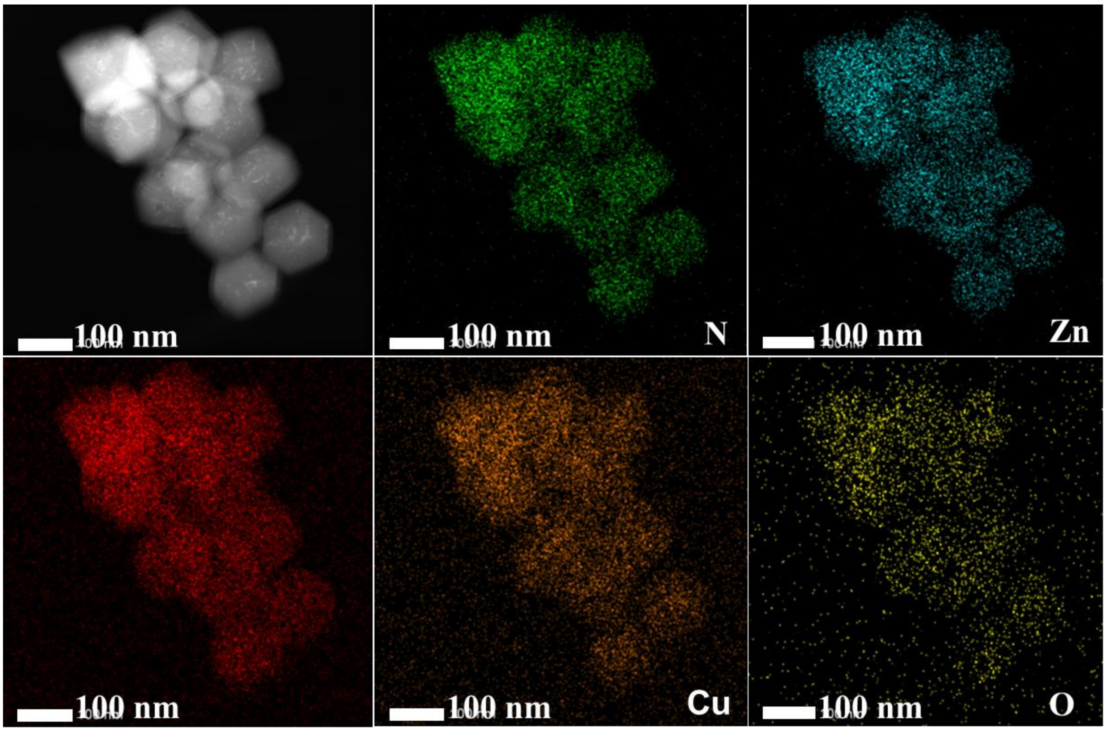

Figure S2. EDS mapping element analysis diagram of CP/ZIF-8

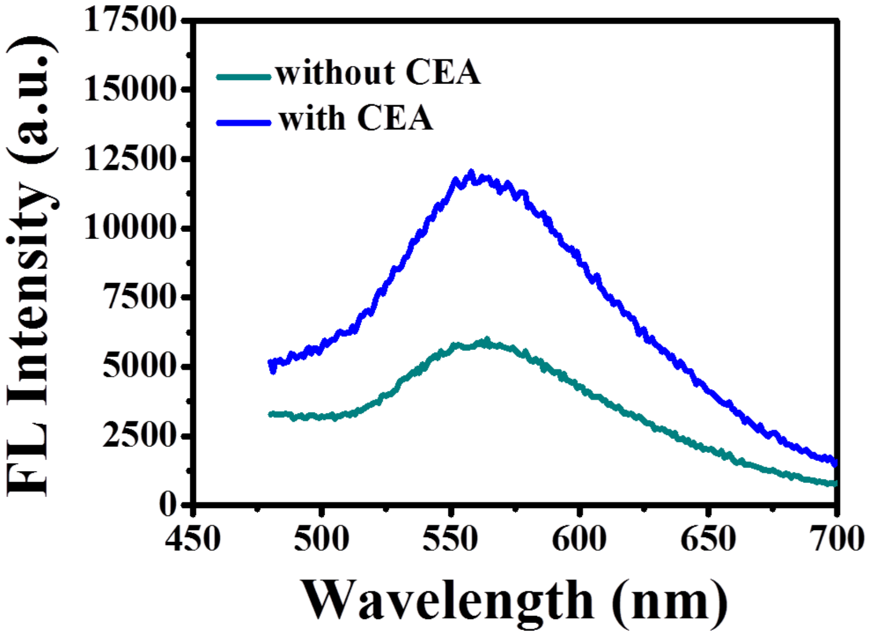

Figure S3. Fluorescence intensity of FLISA platform with and without CEA.

Table S1. Comparison of analytical performance for CEA determination by using different sensing methods.

Method	Recognization/Sensing elements	Linear range (ng mL^{-1})	Limit of detection (pg mL^{-1})	Reference
Electrochemical	Concanavalin/ANCMTs@Fe_3O_4@Cusilicate	0.03-6	5.38	[1]
Electrochemiluminescence	Antibody/Co_3O_4NRs/luminol	0.005-500	2.9	[2]
Scanning electron microscope	Antibody/gold nanoparticle	0.1-80	30	[3]
pH-Based immunoassay	Antibody/glucose oxidase loaded gold hollow microsphere	0.1-100	62	[4]
Fluorescence	Antibody/alkaline phosphatase	0.25-30	80	[5]
Fluorescence	Aptamer/Texas Red/molybdenum disulfide	0.1-100	10	[6]
Fluorescence	Aptamer/glucose loaded mesoporous silica nanocontainers/CdTe/CdSe QDs	0.05-20	6.7	[7]
Fluorescence	CuO_2/ZIF-8/polydopamine / Aptamer	0.01-20	7.6	This work

References

1. Zheng, J.; Wang, J.P.; Song, D.D.; Xu, J.L.; Zhang, M. Electrochemical aptasensor of carcinoembryonic antigen based on concanavalin a-functionalized magnetic copper silicate carbon microtubes and gold-nanocluster-assisted signal amplification. *ACS Appl. Nano Mater.* **2020**, *3*, 3449-3458. https://doi.org/10.1021/acsanm.0c00194
2. Tang, Y.; Zhang, B.H.; Wang, Y.; Zhao, F.Q.; Zeng, B.Z. Electrochemiluminescence immunosensor for the detection of carcinoembryonic antigen based on oxygen vacancy-rich Co3O4 nanorods and luminol. *ACS Appl. Nano Mater.* **2021**, *4*, 7264-7271. https://doi.org/10.1021/acsanm.1c01240
3. Zhou, X.; Yang, C.T.; Xu, Q.S.; Lou, Z.C.; Xu, Z.F.; Thierry, B.; Gu, N. Gold nanoparticle probe-assisted antigen-counting chip using SEM. *ACS Appl. Mater. Interfaces.* **2019**, *11*, 6769-6776. https://doi.org/10.1021/acsami.8b19055
4. Jiang, Y.; Su, Z.Y.; Zhang, J.; Cai, M.J.; Wu, L.L. A novel electrochemical immunoassay for carcinoembryonic antigen based on glucose oxidase-encapsulated nanogold hollow spheres with a pH meter readout. *Analyst* **2018**, *143*, 5271-5277. https://doi.org/10.1039/C8AN01436A
5. Fan, Y.C.; Lv, M.M.; Xue, Y.; Li, J.; Wang, E.K. In situ fluorogenic reaction generated via ascorbic acid for the construction of universal sensing platform. *Anal. Chem.* **2021**, *93*, 6873-6880. https://doi.org/10.1021/acs.analchem.1c00967
6. Xu, S.H.; Feng, X.Y.; Gao, T.; Liu, G.F.; Mao, Y.N.; Lin, J.H.; Yu, X.J.; Luo, X.L. Aptamer induced multicoloured Au NCs-MoS2 "switch on" fluorescence resonance energy transfer biosensor for dual color simultaneous detection of multiple tumor markers by single wavelength excitation. *Anal. Chim. Acta* **2017**, *983*, 173-180. https://doi.org/10.1016/j.aca.2017.06.023
7. Qiu, Z.L.; Shu, J.; Tang, D.P. Bioresponsive release system for visual fluorescence detection of carcinoembryonic antigen from mesoporous silica nanocontainers mediated optical color on quantum dot-enzyme-impregnated paper. *Anal. Chem.* **2017**, *89*, 5152-5160. https://doi.org/10.1021/acs.analchem.7b00989
